# The Role of Choice-Lock Catheter and Trocar Technique in Percutaneous Ablation of Symptomatic Renal Cysts

**DOI:** 10.5812/iranjradiol.16327

**Published:** 2014-05-15

**Authors:** Burak Ozkan, Ali Harman, Baris Emiroglu, Ilker Arer, Cuneyt Aytekin

**Affiliations:** 1 Department of Interventional Radiology, School of Medicine, Baskent University, Besevler, Turkey

**Keywords:** Simple Renal Cysts, Percutaneous Cyst Ablation, Ethanol Sclerotherapy, Choice-Lock Catheter, Trocar Technique

## Abstract

**Background:**

The most common benign lesions of the kidney are simple cysts. They are acquired lesions and mostly affect the elderly population.

**Objectives:**

To describe the usage of choice-lock catheter and trocar technique in percutaneous renal cyst treatment and determining long-term outcomes.

**Patients and Methods:**

This retrospective study was carried out between February 2000 and July 2011. Eighty-eight cysts all of which were Bosniak type-1 cysts were selected in 75 patients. The treatment indications were flank pain, hydronephrosis and hypertension. The choice-lock catheter was used for 84 cysts with the trocar technique. Ninety-five percent ethanol was used as the sclerosing agent. Maximum volume of the injected ethanol was 175 ml. The mean follow-up time after the treatment procedure was 23 months. Sixty-four cysts were located in the cortical and 24 cysts were located at the parapelvic region.

**Results:**

Fifty-seven cysts had complete regression, while 31 cysts regressed partially. After the procedure, pain was relieved in 44 (82%) patients and the pain alleviated in four (8%). Normotension was obtained in five (62.5%) of the eight hypertensive patients and no hydronephrosis was detected in nine patients. There were no relationship between the localization and the regression rate. No major complications occurred.

**Conclusions:**

Percutaneous ethanol sclerotheraphy in simple cysts is a safe, cost-effective and minimally invasive method. We consider that this technique may be an alternative solution in the percutaneous cyst treatment.

## 1. Background

The most common benign lesions of the kidney are the simple cysts. They are acquired lesions that mostly affect the elderly population (1). The incidence of the simple renal cyst is more than 50% at age of 50 years. The renal cysts occur by weakening of the epithelium of the collecting duct cells leading to the diverticula formation process by time (2). The indication for treatment is based on urinary tract obstruction. Several methods for treatment of the cyst including surgical and percutaneous procedures have been proposed; namely, percutaneous marsupialization (3), percutaneous aspiration (4), and open and laparoscopic cyst unroofing (5). Aspiration alone without injection of a sclerosing agent has been reported with a higher recurrence rate. There are several promising results with single or multi-session sclerotherapy with percutaneous drainage (6, 7).

**Figure 1.**
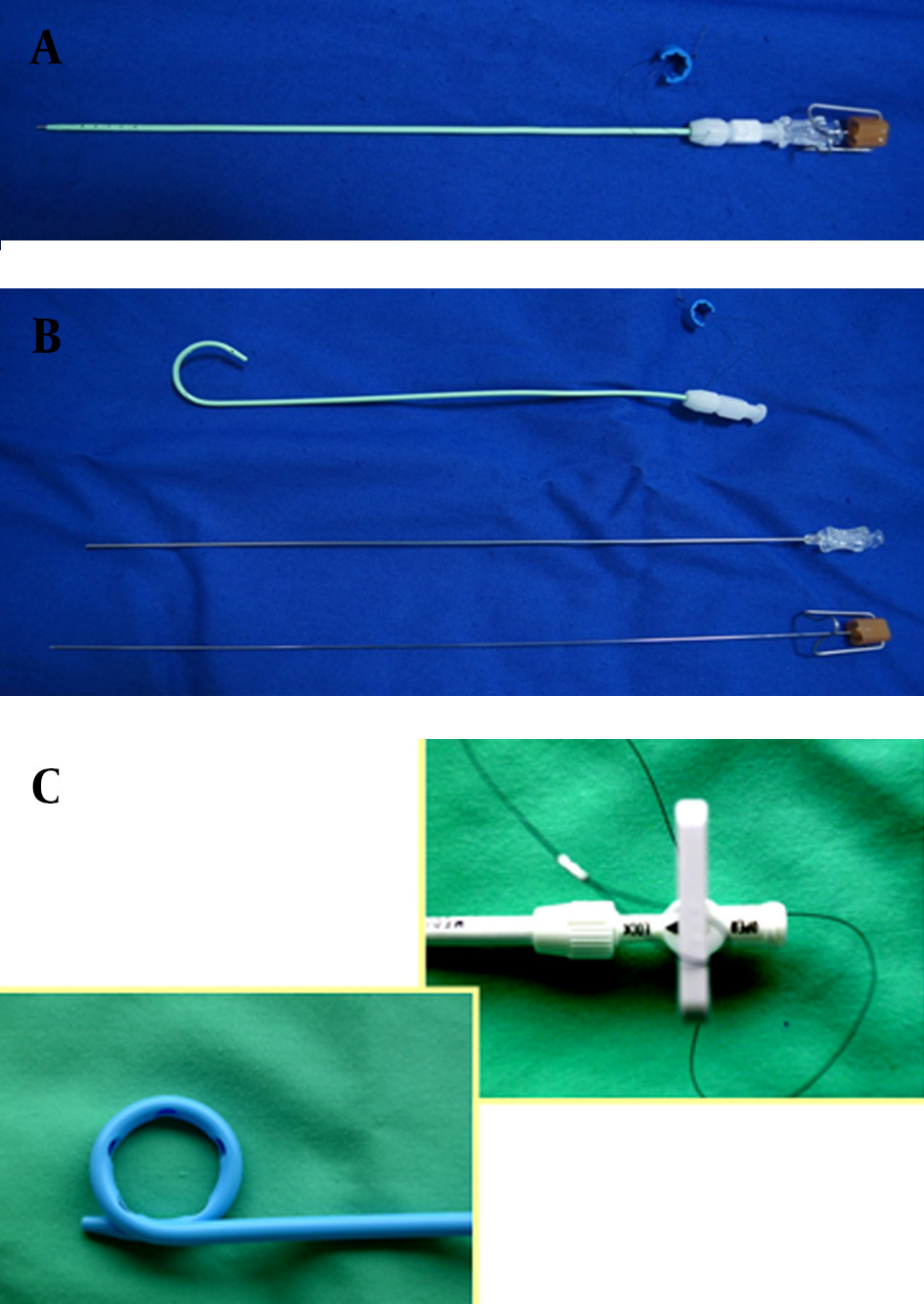
The structure of choice-lock catheter; A) The straight structure of the choice-lock catheter; B) The three co-axial parts of the choice-lock catheter; C) The specific lockage system of the choice-lock catheter

The advantage of choice-lock catheter is the 5.7 F diameter. The diameter of the choice-lock catheter is smaller than other draining catheters. Moreover, other draining catheters are over 6 French sized. Choice-lock catheter consists of three co-axial parts. The stillet, the most inner part of the catheter can also be used for the initial puncture site. This catheter has a special lockage system that enables the catheter stability of the trocar during the puncture with one hand usage. There is a metal cannula for supportive aim between the pigtail plastic catheter and the stillet. The supportive metallic part of the catheter is located in the catheter, it stables the other parts to be guided. This option enables the catheter to be guided safely throughout the hardened tissues. At the most outer part, there is a hydrophilic covered plastic part that has a pigtail lockage system (Figure 1). The catheter can be used instead by the Seldinger technique with the help of 0.018 inch guide wire.

## 2. Objectives

The aim of this study was to assess the role of choice-lock catheter and trocar technique in percutaneous renal cyst treatment.

## 3. Patients and Methods

This retrospective study consisted of 132 patients with 138 simple renal cysts who underwent percutaneous sclerotherapy and ethanol ablation treatment between February 2000 and July 2011 in our clinic. Fifty-seven patients were excluded from the study due to lack of sufficient clinical data especially follow-up data. Most of these patients were referred to our clinic from another hospital. Seventy-five patients with 88 cysts had initial criteria for the study. Forty-two patients were male and 33 were female. The mean age of the patients was 64 years (range, 44-87 years). The patients who underwent percutaneous treatment had only type I Bosniak cysts (8). Sclerotherapy indications included flank pain in 58 (77%), hydronephrosis in nine (10%), and hypertension in eight (9%) patients. The initial criteria for success after treatment was 60% volume reduction of the cyst. Sixty to eighty percent reduction of the cyst volume was considered as partial regression, and more than 80% was considered as complete regression. The choice-lock 5.7 F catheter with trocar technique is used for percutaneous cyst treatment and ethanol sclerotherapy. In four patients, the cysts were septated and aspiration did not yield acceptable treatment results at the first attempt. In these patients, the choice-lock catheter was replaced by 8F pigtail catheter without doing a new puncture. In these cases, subsequently, the 6F dilatator was replaced by the puncture site with the guidance of 0.018 inch wire that was inside the choice-lock catheter. The 8 F pigtail catheter (Flexima, APDL, Boston Scientific, USA) was inserted into the cavity over a 0.035 inch guide wire (Amplatz stiff guide wire, Boston Scientific, USA). Twenty milliliter of the fluid was aspirated from the cavity with the help of the needle. The fluid was checked for further examinations. To obtain the cystogram under fluoroscopy, 50% diluted Telebrix (350 mg iodine/mL, Guerbet, France) was used. The aim of the cystogram is to assess the relationship of the cyst with the collecting system and to determine Bosniak classification. Without any extravasation or communication with the collecting system, the cyst volume was aspirated. Equal to 30% of the initial cyst volume of 95% ethanol was injected into the cavity under fluoroscopic guidance. The patient was checked for any compliance of pain or other related symptoms--if the patient tolerated the session well, 95% ethanol was left in the cavity for 15 minutes. The patient was placed in at least three supine, prone and both lateral decubital positions. The reason was to allow contact of ethanol with all the cyst’s walls in order to destroy the epithelial tissue of the renal cyst. At the end of the procedure, all the injected ethanol to the cavity was aspirated and the catheter was withdrawn. The patients were called for periodic ultrasound and/or CT examinations at several follow-up times. The patients were questioned about the symptoms and the volume of the treated cysts was calculated. The mean follow-up period was 23 months ranging from 3 to 58 months. Forty-two patients were followed for at least 2 years or more (29 patients had 2, six patients had 3, five patients had 4, and two patients had 6 years follow-up).

SPSS for Windows Ver 11.5 (SPSS Inc., Chicago, Ill, USA) was used for statistical analysis. The Shapiro-Wilk test was used to determine if the distribution of the continuous variables were normal. The descriptive statistics for continuous variables were defined as mean±standard deviation or median (min-max). For the categorical variables, the percent of patients and variables was calculated. The Wilcoxon sign rank test was used for evaluation if there was a statistically significant change in the cyst volume before and after treatment. P value less than 0.05 was considered statistically significant. The Hospital Research Ethic Committee approved the study protocol.

## 4. Results

Between February 2000 and June 2011, 88 cysts among 75 patients who had percutaneous cyst aspiration and ethanol sclerotherapy were evaluated. Eighty-four cysts were treated with choice-lock with trocar technique. In four patients, the cysts were septated and aspiration did not yield acceptable results without performing a new puncture; therefore, choice-lock catheter was changed with 8F pigtail catheter under fluoroscopic or sonographic guidance. All catheterization procedures were technically successful. The mean volume of the cyst before treatment was 145.65 mL (39-504 mL) and it reached 15.5 mL (0-126 mL) after treatment (P<0.001). After the procedure, 57 cysts showed a higher than 80% volume reduction and 31 cysts had 60-80% volume reduction. None of the patients had any malignant cells in the cytological examinations.

Sixty-four cysts were located in the cortical and 24 cysts were located at the parapelvic region. Before starting the procedure, the median value of the cyst was 174.8 mL (49-504) ml in the cortical group, and 85 ml (36-175 mL) in the parapelvic group. After the procedure, the median value of the cyst volume was 17.3 mL (0.00-105 mL) in the cortical region group, and 6.8 ml (0-65 mL) in the parapelvic located group. There was no statistically significant difference in the rate of regression between cortical and parapelvic located cysts (P=0.892). There was no statistically significant difference in the rate of regression between two genders and two groups of small and large volume cysts (Tables 1 and 2). A total of 75 patients who underwent percutaneous aspiration and sclerotherapy had no major complications such as renal parenchymal injury, renovascular or renal collecting system injury, pneumothorax or mortality. Patients using ethanol during sclerotherapy can have minor complications such as allergic reaction, microscopic hematuria or infection. Depending on the amount of ethanol used during sclerotherapy patients may develop transient pain and this is the restriction of ethanol usage. This is usually related with ethanol extravasation. No patient had retroperitoneal hemorrhage or hemorrhage into the cyst cavity.

**Table 1.** Mean Volume of the Cysts Before and After Treatment in Two Genders

Gender	Number of Patients (n = 75)	Number of Cysts (n = 88)	The Mean Volume of the Cysts Before Treatment, cc	The Mean Volume of the Cysts After Treatment, cc
**Male**	42	51	146 (39-483)	16 (0-126)
**Female**	33	37	142 (27-504)	15 (0-70)

**Table 2.** Mean Volume of the Cysts Before and After Treatment in Two Groups of Large and Small Cysts

	The Mean Volume of the Cysts Before Treatment, cc	The Mean Volume of the Cysts After Treatment, cc
**Cysts Larger than 300 cc (n = 20)**	330 (309- 504 )	26 (0-70)
**Cysts Smaller than 300 cc (n = 68)**	128.65 (39.6-294 )	14.1 (0-126)

**Figure 2.**
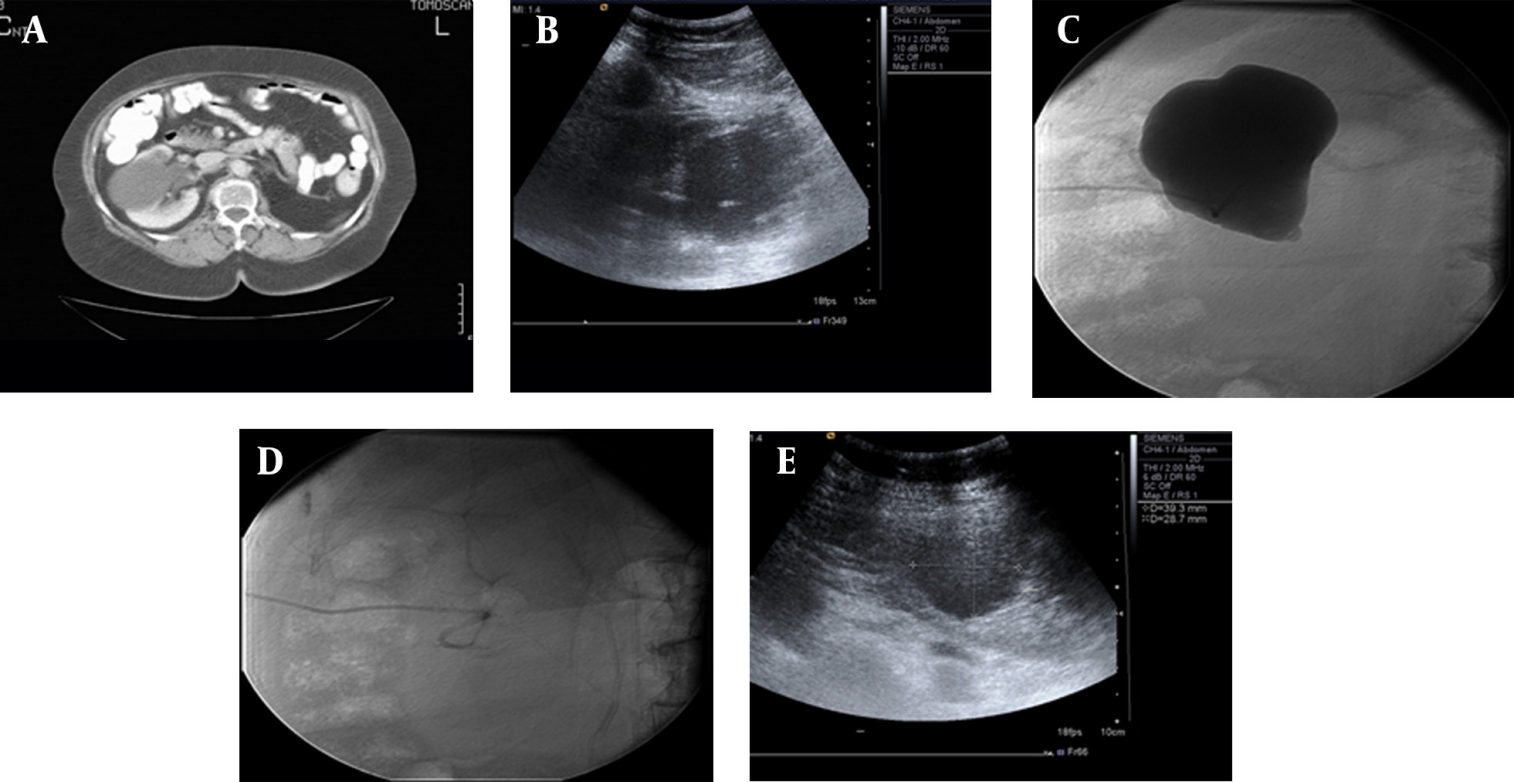
A 56-year-old man with Bosniak type-1 cyst in the right kidney with flank pain. A) Axial CT image shows Bosniak type-1 cyst in the right kidney which has affected the pelvicalyceal system; B) US image shows the initial catheterization of the choice-lock catheter in the cyst during the treatment procedure; C) Cystogram shows that the cyst does not have any relationship with the pelvicalyceal system; D) Cystogram obtained after emptying of the cystic fluid; E) US image shows partial regression of the cyst one month after treatment.

**Figure 3.**
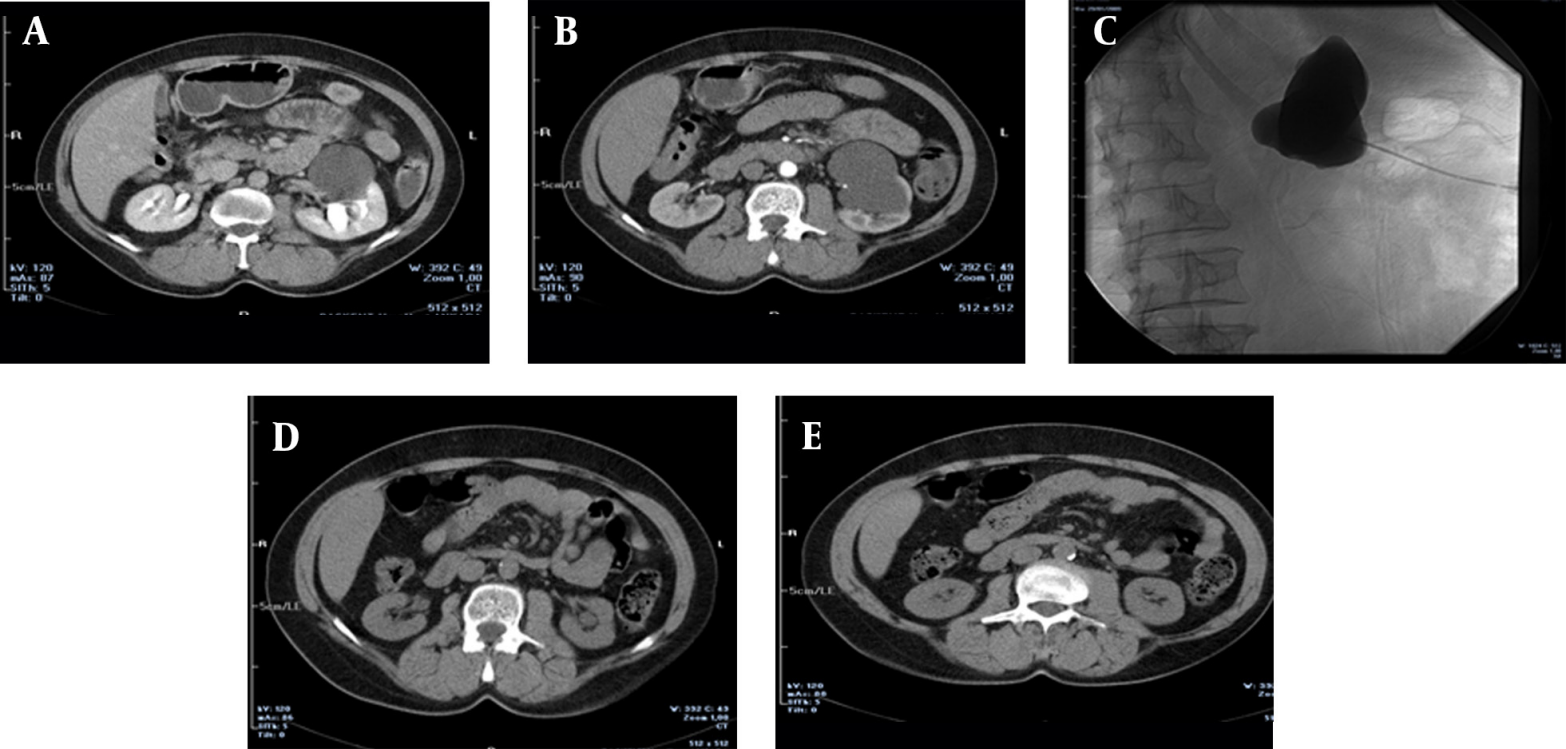
A 72-year-old man suffering from left flank pain for 6 months, A and B) IV contrast enhanced CT images show the cyst located at the lower portion of the left kidney which is classified at Bosniak type-1; C) Cystogram image shows that the cyst does not connect with the pelvicalyceal system; D and E) CT images obtained 3 months after the treatment show that the cyst is completely regressed and the patient had no pain.

Symptoms (flank pain, hydronephrosis and hypertension) resolved in 66 (88%) of 75 symptomatic patients. Forty-eight (83%) of 58 patients with pain responded well to the treatment. Forty-four (76%) were free of pain, in four (6%) patients the pain decreased, whereas in four (6%) patients the pain did not change, and in six (10%) patients, the pain increased. The patients’ symptoms before and after effective sclerotherapy are mentioned in Table 3. The successful treatment of a renal cyst is shown in Figure 2 and Figure 3.

**Table 3.** Patients’ Symptoms Before and After Effective Sclerotherapy

Symptoms	Number of Patients Who Described Symptoms Before Effective Sclerotherapy	Number of Patients Whose Symptoms Resolved After Effective Sclerotherapy
**Flank Pain**	58	44
**Hydronephrosis**	8	5
**Hypertension**	9	8
**Total**	75	57

## 5. Discussion

Percutaneous aspiration and sclerotherapy is the first-line treatment option for symptomatic simple renal cysts (1). Percutaneous aspiration is a simple, safe and minimally invasive procedure. Simple drainage without sclerotherapy is associated with a high recurrence rate of 40-80%. Percutaneous sclerotherapy using a sclerosing agent provides more satisfactory results than aspiration alone (2). In aspiration alone, the destruction of epithelial cell lining will not happen and the epithelial cells will continue secreting fluid cyst. In other words the cyst fluid re-accumulates (4). Secretory epithelial lining the cyst wall must be destructed in order to prevent recurrence. For this purpose, various sclerosing agents such as bismuth-phosphate (9), tetracyclin hydrochloride (10), acetic acid (11), povidone-iodine (12), n-butyl cyanoacrylate and iodized oil (13), ethanolamine oleate (14), OK-432 (15) and minocycline hydrochloride (16) have been used after the cyst fluid aspiration. Ethanol is the most commonly used sclerosing agent. Chemical properties of ethanol, leading to necrosis of epithelial cells lining the cyst wall will produce more obstacles. Secretory cells are rapidly inactivated by ethanol, but penetration of the fibrous capsule takes four to twelve hours. In this way, destruction of cysts occur without affecting the renal parenchyma. Ethanol as a sclerosing agent has been mentioned in the literature, and there are studies that have reported success rates of over 90% (1, 2, 4). Akinci and colleagues treated 98 simple renal cysts with percutaneous ethanol sclerotherapy with a single session technique (2).

At the end of the first year follow-up, the reduction rate in cyst volume was 93.1%. In 17 patients, the cyst disappeared completely, and 83% of the patients had clinical improvement in the symptoms (2). Zerem et al. (17) treated 85 patients and 92 cysts with percutaneous ethanol sclerotherapy. Recurrence of only six cysts occurred at the 24-month follow-up. Mohsen et al. (18) treated 64 cysts of 60 patients using sclerotherapy with 95% ethanol. In 84% the method provided complete resolution. In our study, percutaneous cyst aspiration and ethanol sclerotherapy of 88 cysts was applied. Reduction in the size of the cyst occurred in all 75 patients and 88 cysts after the procedure. In 57 cysts, 80% reduction took place in the volume of the cyst after the procedure out of which 31 had a 60-80% decrease in size. Percutaneous ethanol sclerotherapy in the treatment of symptomatic simple renal cysts were considered as successful (P < 0.001). Our success rate was similar to other studies. In the study conducted by Ozgur et al. (4), a number of patients developed recurrence during follow-up while a sclerosing agent was not used for any of them. In our study, no recurrence or increase in the cyst volume was reported during follow-up. We think that this is due to the usage of ethanol as the sclerosing agent.

The single session of sclerotherapy with ethanol in the literature reported high success rates. There are also studies indicating that multiple session sclerotherapy is a more effective method of treatment. Hanna and Dahniya have shown increased success rates after two sessions of ethanol sclerotherapy. The recurrence rate was 80% in the group on which only aspiration was carried out and 32% in the group on which percutaneous aspiration and single-session ethanol therapy was performed. No recurrence occurred in the group on which ethanol sclerotherapy was carried out twice. The high success rate of sclerotherapy with ethanol depends on the injection by increasing the amount of contact time (7). Fontana and colleagues used the three-time ethanol injection method. The amount of ethanol used in the treatment was up to 30% of the volume and did not exceed 60 ml per each cyst treatment. Free drainage method of the cyst was used. As a result, recurrence of the cyst occurred in two patients and 68 cysts had complete resolution. The higher success rate in this study was based on the higher ethanol concentration at the cyst wall. The higher ethanol concentration results in the greater destruction of the epithelial tissue. Ethanol penetrates the fibrous capsule slowly that is important in preventing systemic complication (19).

There were limitations in this study. Four of the 88 cysts had treatment with 8F caliber catheter. The number of patients was not enough for the comparison. In other words, there was not enough data to compare the choice-lock catheter and the 8F catheter. The second limitation was the pain scoring system. This is a retrospective study and we did not have a scoring system for the treatment outcome related to pain. But now in our clinic we use the pain scoring system and check before and after the procedure.

In conclusion, percutaneous aspiration and ethanol sclerotherapy is an effective way of treatment of simple cysts. Our study is the first paper that describes the usage of trocar technique and choice-lock catheter.
